# Enzymatically Interesterified *Triadica sebifera* Oil: A Novel Shortening for Enhanced Nutritional Quality and Sustainability

**DOI:** 10.3390/foods14040590

**Published:** 2025-02-11

**Authors:** Ying Liu, Pan Gao, Yong Yang, Chunxiao Liu, Wu Zhong, Jiaojiao Yin, Martin J. T. Reaney

**Affiliations:** 1 Key Laboratory of Edible Oil Quality and Safety, State Administration for Market Regulation, Key Laboratory for Deep Processing of Major Grain and Oil of Ministry of Education in China, College of Food Science and Engineering, Wuhan Polytechnic University, Wuhan 430023, China; 2 Wuhan Institute for Food and Cosmetic Control, Wuhan 430012, China; 3 Department of Food Science, University of Saskatchewan, Saskatoon, SK S7N 5A8, Canada

**Keywords:** *Triadica sebifera* oil, soybean oil, enzymatic interesterification, shortening, healthy nutrition

## Abstract

Trans-fatty acids (TFAs) in conventional shortenings are associated with health concerns. Here, we explore approaches to produce a healthier alternative using *Triadica sebifera* oil (TSO) and soybean oil (SO). Through optimized enzymatic interesterification (EIE), a 6:4 TSO to SO ratio was used, with a reaction temperature of 60 °C, enzyme dosage of 2%, and 240 min duration. The resulting product was free of TFAs, rich in unsaturated fatty acids (50.31%), and exhibited desirable physical attributes suitable for commercial shortening. The oil produced through EIE displayed a β′ crystal form and an improved melting profile for better texture. This novel shortening meets commercial standards and can replace commercial shortening with TFA in baking and cooking applications. This substitution would lead to a healthier shortening product. The EIE process requires fewer inputs than hydrogenation and is a more environmentally friendly approach for shortening production. This research contributes to more sustainable practices in the food industry while offering a practical solution for healthier shortening.

## 1. Introduction

*Triadica sebifera* (TS) is one of China’s four predominant woody oilseed species, with 20,000 tons produced in 2022. *Triadica sebifera* oil (TSO) is extracted from the external white waxy layer of TS seeds. TSO is non-toxic, edible, and has emerged as a versatile candidate for diverse applications owing to its abundant content of palmitic acid (C16:0) and desirable solid fat characteristics [1,2]. Extensive research has been directed toward the use of TSO as a resource for producing environmentally friendly biodiesel, but its potential within the food industry remains largely unexplored. This resource has not been fully utilized in food applications [3,4,5,6]. As a result of reduced production and distribution costs, palm oil (PO) has become a functional ingredient in many common shortenings. However, the expansion of palm plantations and the resulting deforestation raise concerns related to sustainability and carbon emissions [7]. Here, TSO presents an environmentally sustainable alternative, boasting a high content of palmitic acid (C16:0) and an optimal solid fat content (SFC), making it a viable substitute for PO-based shortenings [8].

As consumer demand for premium food quality and flavor intensifies, coupled with a penchant for crisp and savory dishes, the demand for healthier shortening alternatives is increasing. However, most available shortenings are typically manufactured through physical blending processes, which unfortunately leads to products with compromised safety, reduced nutritional value, and heightened levels of saturated fatty acids (SFA) and trans-fatty acids (TFA). The consumption of such shortenings poses a heightened risk of chronic diseases, including cardiovascular disease and obesity [9,10]. To address this, enzymatic interesterification (EIE) is emerging as a healthier alternative to conventional hydrogenation, enabling the production of shortening that is both nutritious and environmentally friendly [11]. Unlike hydrogenation, which can produce TFA, enzymatic interesterification enables the structural modification of fats without generating TFAs. This technology affords the preservation of TSO’s natural antioxidants and trace minerals, enhancing the shortening’s health benefits [12]. Lipases play an important role in EIE, and several commercial lipases (Lipozyme TL IM, Lipozyme RM IM, and Novozym 435) have been extensively studied [13]. The choice of enzyme is critical for EIE. Particularly, Lipozyme TL IM is a leading candidate due to its high thermal stability and efficient catalysis [14]. As such, it is suitable for industrial applications that minimize environmental pollution. Nevertheless, TSO’s inherent hardness and high melting point could negatively affect the shortening’s flavor and texture. Soybean oil (SO) has good lubricity and composition, which provides health benefits. The inclusion of SO in shortening might mitigate the negative effects on texture while enhancing both the taste and healthfulness of the shortening.

This study aims to explore EIE technologies as a method to prepare blends of TSO and SO and to evaluate the blends for use as a new shortening that might help fulfill the growing demand for sustainable and nutritious food products. The use of TSO in the food industry would help in resource diversification and environmental stewardship. With Lipozyme TL IM identified as the catalyst, this research explores the optimal conditions for EIE to produce TSO-based shortening with enhanced nutritional and texture properties, promising a healthier and environmentally friendly approach to fat product preparation for the food industry.

## 2. Materials and Methods

### 2.1. Materials

TSO was procured from Shuyang Donghu Oil Co., Ltd., located in Shuyang, Jiangsu, China. SO and PO were obtained from Yihai Kerry Oil & Co., Ltd., Wuhan, Hubei, China. The enzymatic catalyst Lipozyme TL IM, a commercial immobilized lipase from a strain of *Thermomyces lanuginosus*, was purchased from Novozymes Biotechnology Co., Ltd. (Yueyang, Hunan, China). The standards of fatty acid methyl ester and tocopherol (purity ≥ 95%) were purchased from Sigma-Aldrich Chemical Co., Ltd. (Shanghai, China). n-Hexane, anhydrous ethanol, and other reagents were purchased from Sinopharm Chemical Reagent Co., Ltd. (Wuhan, Hubei, China). For comparative analysis, commercial shortening samples labeled A, B, and C were obtained from a local manufacturer in China.

### 2.2. Preparation of TSO-Based Shortening

TSO-based shortening was prepared according to the method described by Xu et al., [15], with some modifications. Initially, the solid lipid TSO was melted at 70 °C for 3 min. Subsequently, varying mass ratios of TSO to SO (1:9 to 9:1, with a total substrate weight of 50 g) were combined in 100 mL conical flasks with screw caps. Lipozyme TL IM was used as the biocatalyst at concentrations ranging from 0% to 2.5% *w*/*w*. The reaction mixture was then incubated in a thermostatic water bath at 50–70 °C with stirring at 350 rpm for durations ranging from 1 to 5 h. Upon reaction completion, the mixture was centrifuged at 14,000 rpm for 2 min to remove the catalyst, and the filtrate was collected. This filtrate was subsequently washed with warm water at 60 °C until neutral and dehydrated under a vacuum of 0.1 MPa for 30 min to yield the final shortening product.

### 2.3. Physicochemical Properties

The determination of acid value (AV) and peroxide value (PV) follows the laboratory method: in AV determination, 1.0 g of oil sample was weighed, 50 mL of a mixture of ether and isopropanol (1:1) was added, phenolphthalein indicator was added dropwise, and 0.01 mol/L NaOH standard solution was titrated until it turned red. In the PV measurement, 2.0 g of oil sample was weighed, 30 mL of a mixture of chloroform and glacial acetic acid (2:3) was added, 1 mL of saturated potassium iodide solution was added, shaken well, and left in the dark for 3 min. Then, 100 mL of distilled water and 1 mL of starch indicator were added and titrated with 0.01 mol/L sodium thiosulfate standard solution until the blue color disappeared. Water content was assessed by heating a 5.0 g sample of shortening at 103 ± 2 °C until a constant weight was achieved. The oxidation stability index (OSI) was determined based on the procedure outlined in our previous paper [16]. Next, 3.00 g of oil sample was accurately weighed into a specialized test tube for the Hairizo Rancimat instrument and set to a temperature of 120.0 °C and an intake flow rate of 20.0 L/h. Polyphenol content was quantified using the Folin–Ciocalteu method [17,18]. The whipping performance of the shortening samples used a previously established method [19], where samples were preconditioned at 20 °C for 24 h. For testing, 500 g of the sample was whisked at speed setting to 1 for 30 s, repeated once, before increasing the speed to 5. The whipped samples were collected every 5 min, flattened in a weighing cup, and weighed (Wt). Whipping performance was calculated as (Wt − W_0_)/W, where W_0_ represents the initial weight of the empty weighing cup and W is the combined weight of water and the weighing cup.

### 2.4. Fatty Acid Composition

Samples (0.2 g) were combined with 2 mL of a 0.5 mol/L sodium hydroxide-methanol solution and incubated at 65 °C for 30 min. Subsequently, 8 mL of a 0.1 mol/L NaHSO_4_ solution was added to neutralize excess KOH. The mixture was then heated in a water bath at 65 °C for 3 min before adding 2 mL of chromatographically pure n-hexane. After vortexing and shaking for 1 min, the mixture was allowed to stand for 5 min to facilitate stratification. The upper layer was filtered through a 0.22 μm membrane for gas chromatography-mass spectrometry analysis using an Agilent™ 1260 system (Palo Alto, CA, USA). The analytical conditions included an HP-FFAP column (30 m × 0.25 mm × 0.25 μm, Agilent, Santa Clara, CA, USA) with a flow rate of helium at 1.0 mL/min and a temperature gradient starting at 140 °C, rising to 176 °C at 5 °C/min, and then to 180 °C at 1 °C/min, before being held for 5 min. The injection volume was 1.0 µL with a bypass ratio of 1:30. Mass spectrometry settings were as follows: EI ion source, electron energy of 70.0 eV, ion source temperature of 230 °C, and a scan range of 50.0~550.0 u for a full scan. Fatty acid content was quantified using the response factor combined with normalizing peak areas [15].

### 2.5. The Slip Melting Point (SMP) and X-Ray Diffraction (XRD)

SMP measurements were performed using the AOCS official method Cc 1–25. XRD analysis was conducted based on the procedure outlined in our previous paper [18]. XRD analysis provided insights into the crystalline structures of the samples. XRD analysis was performed using a Bruker GmbH D8 Phaser X-ray diffractometer (Billerica, MA, USA) at 25 °C. The X-rays were generated using a Cu tube operating at 30 mA and 40 kV. Samples were scanned over angular ranges from 5° to 40°at a rate of 4° per minute and a step size of 0.01°. The transmitting and anti-transmitting slits were both set to a width of 1.0 mm, while the receiving slit width was adjusted to 0.1 mm. Data analysis was carried out using MDI Jade 5.0 software from Materials Data Inc., Livermore, CA, USA.

### 2.6. Differential Scanning Calorimetry (DSC) and Solid Fat Content (SFC)

DSC analysis involved encapsulating 6–8 mg of the samples in aluminum discs, with blank discs serving as controls. The samples were heated to 80 °C at 10 °C/min, maintained for 10 min, then cooled to −30 °C at 5 °C/min, and held for another 10 min. This process was repeated with continuous heating to 80 °C at 5 °C/min to analyze the melting behavior [20].

SFC determination was conducted using a Maran SFC (MQC, Oxford, UK) nuclear magnetic resonance spectrometer (NMR). The samples were prepared in special glass tubes, melted at 100 °C for 10 min to eliminate any crystalline memory, tempered at 60 °C for 10 min, followed by cooling at 0 °C for 60 min. Each measurement temperature involved holding the sample at a stable temperature for 30 min in 5 °C increments, with SFC values recorded accordingly [21]. F1–F9 represent oils with different mass ratios (TSO:SO = 1:9, 2:8, 3:7, 4:6, 5:5, 6:4, 7:3, 8:2, 9:1).

### 2.7. Statistical Analysis

Experiments were conducted three times to ensure reproducibility, with data presented as the mean ± standard deviation. Statistical analysis was performed using SPSS 23.0 (IBM Corp., Armonk, NY, USA), Microsoft Excel, and MATLAB 2022 (Mathworks, Natick, MA, USA), employing Duncan’s test to evaluate all parameters. Origin 2018 software facilitated the plotting and linear fitting analysis of standard curves. Differences were considered statistically significant at the 5% level (*p* < 0.05).

## 3. Results

### 3.1. Solid Fat Content (SFC)

**Figure 1:**
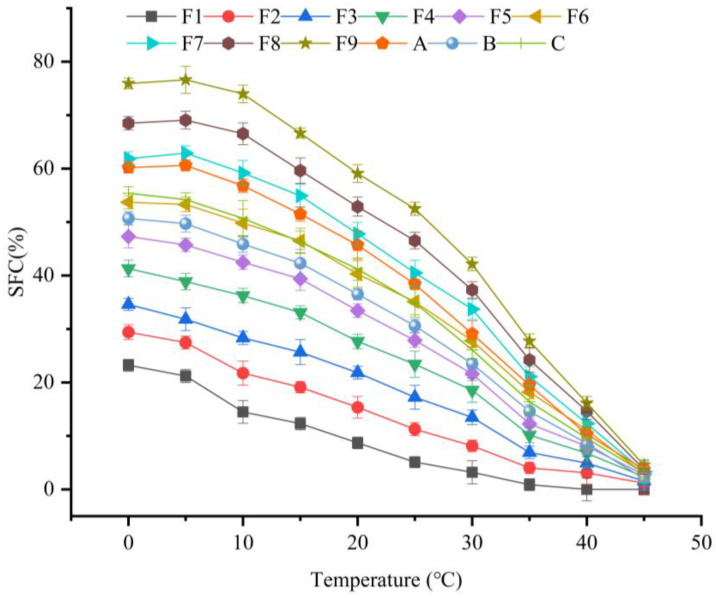
SFC curves after enzymatic transesterification between different ratios of *Triadica sebifera* oil (TSO), soybean oil (SO), and commercially available shortening A, B, and C.

The functional properties of margarine and shortening products, especially spreadability and stability, are largely determined by the SFC. The crystal structure and polycrystallinity of solid fats also influence their performance [22]. Our analysis (Figure 1) compares SFC across various TSO and SO ratios, as well as commercial shortenings A, B, and C. As expected, the SFC for all samples decreased as the temperature approached the respective melting point. Specifically, an ideal spreadability under refrigeration (5–10 °C) was achieved with an SFC of less than 32%. However, except for samples F1, F2, and F3, all other samples exceeded this threshold, indicating a decrease in spreading at lower temperatures. In order to avoid oil seepage in the transport or processing of food fats and oils, it is generally required that the SFC of oils should be greater than 10% at 20 °C. In contrast, F6 demonstrated superior room-temperature stability, with an SFC of 40.3%. This SFC is consistent with commercial standards (36.5–45.7%). Notably, for product F6, the SFC at 35 °C of 18.2% closely matched the exemplary commercial product SFC range of 14.6–19.5%, embodying the characteristics of a high-stability shortening [23]. Among the treatments, F6 (TSO:SO = 6:4) had SFC and SMP properties that were the most appropriate for TSO-based shortening. Investigating EIE under varying conditions revealed distinct impacts on shortening properties.

### 3.2. Optimization of Enzymatic Interesterification

#### 3.2.1. Effect of Reaction Temperature

**Figure 2:**
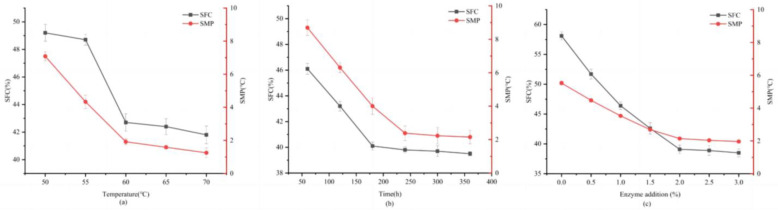
(**a**) Effect of temperature on SFC and SMP. The reaction conditions were 2% catalyst dosage (relative to the weight of all substrates) and 240 min reaction time. (**b**) Effect of reaction time on SFC and MPD of interesterified oils. The reaction conditions were 2% catalyst dosage (relative to the weight of all substrates) at 60 °C. (**c**) Effect of enzyme addition on the SFC and MPD of interesterified oils. The reaction conditions were a reaction time of 240 min at 60 °C.

The effect of the reaction temperature on the SFC and SMP of TSO-based shortening is shown in Figure 2a. The SFC and SMP of the samples gradually decreased with increasing reaction temperature; an increase from 50 °C to 60 °C significantly lowered the SFC by 6.5% and reduced the SMP from 47.5 °C to 41.3 °C. The altered melting point was attained by the induction of lipase activity, which led to the rearrangement of TAGs [24] and the formation of lower-melting substances. However, the negligible decrease in SFC and SMP within the range of 60–70 °C and the high temperature indicate a higher rate of lipase inactivation. Lower reaction temperatures reduce non-selective reactions and minimize energy consumption. When compared with higher temperature reactions, lower temperatures reduce greenhouse gas emissions; therefore, an optimal temperature of 60 °C is best suited for balancing enzyme efficiency and sustainable practices.

#### 3.2.2. Effect of Reaction Time

The SFC and SMP showed stabilization at 39.8% and 41.1 °C, respectively, after a reaction time of 240 min (Figure 2b). Beyond this time, the stable performance suggests that equilibrium was achieved. Excessive reaction times increase the degree of oil oxidation. Lipase-catalyzed interesterification is a reversible reaction, which also fully explains why the SFC and SMP of the ester exchange products first decreased and then increased with the reaction time. Treatment for 240 min produced a product with SFC (39.76%) and SMP (41.1 °C) that were similar to commercial shortenings.

#### 3.2.3. Effect of Catalyst Dosage

Increasing the enzyme concentration up to 2% decreased SMP and SFC (19% and 9.3 °C, respectively; Figure 2c). The increased reaction rate was likely proportional to increased TAG-enzyme levels [25]. Beyond 2%, no significant improvements were observed, likely due to the excess enzyme increasing the viscosity of the system, which decreased mass transfer and catalysis efficiency [26]. Consequently, as the amount of enzyme added increases, the additional cost of the EIE process increases. Therefore, a 2% enzyme dosage is recommended for optimal shortening properties.

These findings underscore the importance of carefully controlling the EIE process, with a TSO:SO ratio of 6:4, reaction temperature of 60 °C, enzyme dosage of 2%, and reaction time of 240 min identified as optimal parameters. This comprehensive approach considers efficiency, sustainability, and health implications, laying the groundwork for further experimental validation. The environmental benefits of this shortening include a reduced dependency on PO, contributing to less deforestation and biodiversity loss.

### 3.3. Physicochemical Analysis

The physicochemical properties of TSO-based shortening and their comparison with those of commercial shortenings are detailed in Table 1. The SMP of interesterified oil (IO) was 41.3 °C, aligning closely with commercial products, which ranged from 39.8 to 42.6 °C. Notably, the SMP of IO showed a significant reduction from TSO’s original 51.4 °C to 41.3 °C, indicative of the formation of more asymmetric TAGs. This structural asymmetry contributes to a lower melting point, thereby enhancing the shortening’s texture to closely mimic that of the normal human body temperature [27]. In contrast, the material produced as a physical blend (PB) did not exhibit a detectable SMP at room temperature. This PB product is, therefore, not suitable for use as a shortening. These findings underscore the complexity and solidity of EIE through the creation of medium- and long-chain fatty acid mixtures. This complexity contrasts with the simpler fat composition of PB, which remains liquid at room temperature. The SMP reduction in IO, when compared with TSO, is consistent with the findings from shortening formulations using other oil stocks. These findings reinforce the efficacy of EIE in improving shortening properties [12].

Oil quality parameters of IO, AV, and PV were 0.79 mg/g and 2.80 mmol/kg, respectively, below the threshold set for edible oil, i.e., AV should not exceed 2.5 mg/g, and PV should be less than 0.25 g/100 g [18]. During interesterification, enzyme catalysis was associated with some hydrolysis of ester bonds. In addition, with handling, some oxidation of fatty acids was evident based on the elevated free fatty acid and peroxide content post-interesterification. Despite these findings, the quality parameters of AV and PV were suitable for a commercial product. The water content further affirmed the uniformity of the quality across the samples.

Interestingly, the plasticity range (17.5 °C) and whipping capacity (1.85 mL/g) of IO were comparable with commercial standards. The whipping degree varied from sample to sample, probably due to variations in the content of monoacylglycerols (MAGs) in each sample. Unsaturated MAGs form large fat globules due to agglomeration during churning. The clustered fat globules grow in size and are prone to breaking the interfacial membrane of the bubbles. This results in foam collapse and diminished whipping ability. The close whipping degree of IO to commercial benchmarks highlights its potential to mimic the functional attributes of conventional shortenings while providing a healthier alternative.

The polyphenol content analysis determined by the Folin–Ciocalteu method showed that both the PB (17.32 mg/kg) and IO (17.23 mg/kg) had higher polyphenols than TSO (9.85 mg/kg), suggesting the enzymatic process might preserve or even amplify the antioxidant capacity of PB and IO. There might be two reasons for this phenomenon: The first is a synergistic effect where mixing the polyphenols leads to a more intense reaction with the reagent. Second, the Folin–Ciocalteu reagent can react with other reductants in the oils. These findings advocate for the health benefits of IO, offering a richer nutritional profile alongside functional superiority.

The OSI analysis showed that IO (6.73 h) outperformed PB (5.79 h) in shelf-life stability, contradicting the expectation that unsaturated fat enrichment through EIE would increase susceptibility to oxidation [28]. This paradox could be attributed to the enzymatic process fostering a more resilient oil matrix, thereby enhancing the oxidative stability and potentially extending the utility of TSO-based shortenings in food technology applications [29]. This robustness, coupled with a balanced fatty acid profile, underscores the value of TSO as a bioresource for developing healthier, sustainable shortening alternatives.

### 3.4. Fatty Acid Composition

Table 2 shows the fatty acid profiles of TSO, PO, SO, PB, IO, and commercially available shortening. Predominantly, PO exhibited high levels of C16:0 (58.0%) and C18:1 (25.0%) attributes that lend it solidity at room temperature due to its saturated fatty acid (SFA) richness, making it a candidate for inclusion in margarine and shortening products. The composition of TSO was comparably rich in SFAs (65.53%), providing natural firmness, yet potentially limiting its direct application due to restricted plasticity. In contrast, SO, rich in polyunsaturated fatty acids, especially C18:2 (53.9%), offers benefits for cardiovascular, nerve, and visual health, while enhancing the plasticity of the blend [30].

Upon EIE, the SFA content in both PB shortening and IO was markedly reduced to approximately 49.63% and 49.55%, respectively, aligning with the goal of formulating a product for health-conscious consumers. Notably, some commercial shortenings exhibited detectable TFA levels. A commercial product exceeding 0.30% TFA cannot be labeled as zero TFA. Such products are seen as posing a health risk of coronary heart disease, atherosclerosis, and even cancer [11]. Conversely, neither TSO nor SO, as well as the resultant IO, were TFAs detected in the final product, underscoring the health advantages of the EIE process [26]. These features not only contribute to healthier consumer options but also comply with stringent food safety regulations. An economic analysis reveals that while the initial cost of producing enzymatically interesterified shortening is higher than that of traditional methods, the long-term benefits, such as reduced healthcare costs and potential market premium for healthier products, justify the investment.

### 3.5. Differential Scanning Calorimetry

**Figure 3:**
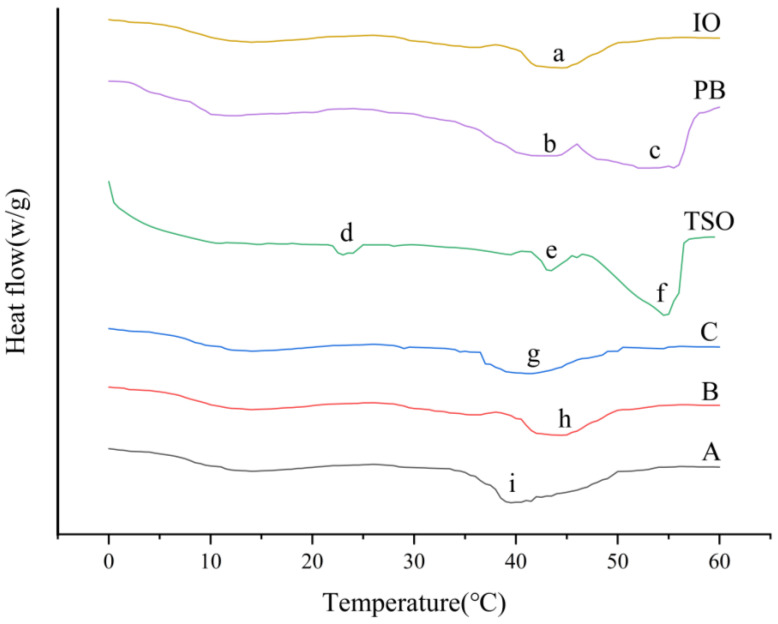
Melting thermograms of TSO, PB, IO, and commercially available shortening A, B, and C.

DSC analysis is visualized in Figure 3, which illustrates the melting behavior of oils, commercial shortenings, PB shortening, and IO. While enthalpy measurements (ΔH) would provide additional insights into thermal transitions, our analysis primarily focuses on the qualitative aspects of the phase transitions, such as peak temperature shifts and melting profiles. IO displayed a more gradual heat-absorption peak at 41.13 °C, indicative of a lower melting point transition compared to PB shortening and closer to the commercially available A. The observed peaks correlate with the crystalline structures present, where the low-temperature peak (around 0–10.0 °C) represents the α form, the mid-temperature peak (around 10.0–50.0 °C) indicates the β′ form, and the high-temperature peak (around 50.0 °C) signifies the β form. IO and PB had two main absorption peaks, b and c. After the EIE reaction, the TSO-based shortening became one main absorption peak a. The post-reaction peaks were a merger of the pre-reaction peak b and peak c. This shift suggests the enrichment of low-melting-point TAG species post-EIE, aligning closely with commercial standards. The absorption peak of IO was smaller than that of PB shortening, which was due to the rearrangement of fatty acids within TAGs or between two TAGs. Compared with the PB shortening, the whole system of IO mainly showed β′ type of crystals. Since β′ type of crystals can produce a tight structure and a smooth and delicate texture, the IO meets the expectation of β′ type of crystals of shortening and can be used as a base oil for inclusion in shortening. In addition, for good shortening, the melting endpoint should be in the range of 39.0 to 45.0 °C. This melting property allows the grease to melt slowly in the mouth, which greatly enhances the taste of the grease [31]. As can be seen from Figure 3, after the EIE reaction, the melting endpoint of IO was 41.27 °C, which was in line with the temperature range of 39.0–45.0 °C. The melting endpoint of PB shortening was 55.42 °C, which was much higher than the specified range. The analysis of the thermal properties of the interesterified oils showed that interesterified oils are beneficial for the production of shortening.

### 3.6. Polymorphism and Fat Crystal Microstructure

XRD analysis provided insights into the polymorphic transitions induced by EIE. Namely, the α (4.15 Å), β (4.50–4.60 Å) and β′ (3.71 Å, 3.80 Å, 3.97 Å, 4.05 Å, 4.20 Å, 4.27 Å) forms are the main polymorphs of lipid. The α-type is the least stable and has the lowest melting point. In contrast, the β-type is the most stable and has the highest melting point. The β′ form is in an intermediate state, both in terms of stability and melting point, and is the best polycrystalline form in terms of shortening [13].

**Figure 4:**
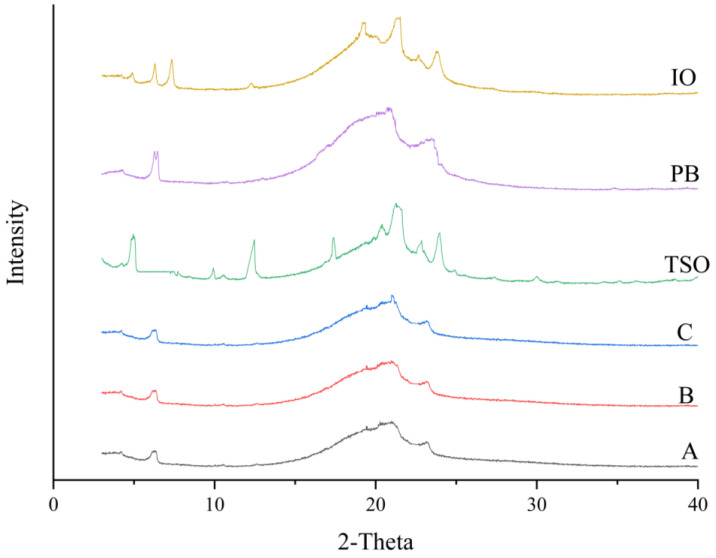
X-ray diffractograms of TSO, PB, IO, and commercially available shortening A, B, and C.

According to Figure 4, it can be seen that PB shortening exhibited β and β′ forms at specific angstroms; the β-type was found at 4.60 Å, and the β′ type at 4.20 Å and 3.80 Å. In contrast, IO facilitated a pronounced shift toward stronger β′ type at 4.20 Å and 3.80 Å, and weaker β′ type at 4.27 Å. This indicates that after EIE, the transformation to a structure is more conducive to shortening its functional requirements. β′ is the most desirable crystal type for shortening. β′ crystals are fine and homogeneous, showing a smooth and soft texture, with a large specific surface area to bind liquid-phase oils, thus preventing phase separation of shortening during storage and providing good stability, gas holding, casualization, and creaming properties [32]. In our study, it was found that the change in crystal type in IO compared to that in PB shortening indicated that the crystalline functional properties of the system were improved after enzymatic transesterification, which was similar to commercially available shortening. The β′ form has a smooth and homogeneous texture that has emerged as the dominant structure in IO, mirroring commercial product characteristics and suggesting enhanced utility in culinary applications.

These findings emphasize the impact of EIE on the physical and chemical properties of shortening, presenting a viable pathway for healthier, functionally superior shortening formulations. The successful modulation of fatty acid composition and crystalline structures through EIE positions this approach as a promising strategy for the development of next-generation shortenings tailored for both health benefits and culinary excellence. The newly developed shortening could be seamlessly integrated into existing food production lines, particularly in baking and frying processes, where it can replace traditional shortenings without altering the end product’s taste or texture.

## 4. Discussion

In this study, we successfully developed a novel shortening method by enzymatically identifying TSO with SO using Lipozyme TL IM as the biocatalyst. This innovative approach utilized a mass ratio of 6:4 (TSO:SO), with SFC serving as a key metric for evaluation. The optimization of reaction conditions—specifically, a temperature of 60.0 °C, an enzyme concentration of 2.0% *w*/*w*, and a duration of 240 min—led to products that aligned with commercial standards for use as a shortening in terms of physicochemical properties, including AV, PV, moisture, odor, SMP, OSI, polyphenol content, plasticity, and whipping performance. Notably, the EIE process facilitated a transformation in the shortening’s crystalline structure from β to the more desirable β′ form, as evidenced by XRD analysis. This structural shift contributes to the shortening’s enhanced textural qualities. Furthermore, fatty acid analysis revealed a significant reduction in SFAs, with the IO containing 50.31% UFAs and no TFAs, highlighting its improved nutritional profile. DSC analysis confirmed that the melting endpoint of the IO falls within the optimal range for shortening (39.0–45.0 °C), underscoring its suitability for culinary applications.

The application of EIE technology in the creation of TSO-based shortening represents a significant advancement, offering an alternative to traditional hydrogenated fats and surpassing the quality of many commercially available shortenings. Such advancements are required to stimulate market demand for TSO, aligning with the growing consumer preference for healthier, more natural food products. However, challenges such as the complexity and cost of the EIE production process, along with environmental considerations related to energy, highlight areas for future improvement. Future research will explore the practical applications of TSO-based shortening in bakery goods and assess its impact on the texture, mouthfeel, shelf life, and nutritional value of products made with this ingredient. This direction not only promises to broaden the utility of TSO in the food industry but also to contribute valuable insights into the development of healthier, more sustainable food processing techniques.

## 5. Conclusions

This study successfully developed trans-fat-free shortening through the enzymatic interesterification (EIE) of *Triadica sebifera* oil (TSO) and soybean oil (SO). By optimizing the reaction conditions, a TSO:SO ratio of 6:4, reaction temperature of 60 °C, enzyme dosage of 2%, and reaction time of 240 min was identified as the most effective formulation. The resulting product demonstrated desirable physicochemical properties that meet commercial shortening standards. The developed shortening offers significant nutritional benefits, containing 50.31% unsaturated fatty acids (UFAs) and no trans-fatty acids (TFAs). This composition makes it a healthier alternative to traditional hydrogenated fats while maintaining essential functional properties. Physicochemical analyses confirmed that the shortening resulted in an optimized melting profile, β′ crystal structure, and stable SFC. These characteristics contribute to improved texture, spreadability, and overall performance in food applications. Additionally, the shortening demonstrated better oxidative stability (OSI) than the physical blends, ensuring a longer shelf life. Beyond its functional and nutritional advantages, the EIE process presents a more sustainable approach for shortening production. By eliminating hydrogenation and reducing reliance on palm oil, this method offers a commercially viable and environmentally friendly alternative for food manufacturers. The optimized formulation is particularly well suited for applications in baking and frying, where texture and stability are critical. This study establishes EIE as an effective and scalable solution for producing high-quality, health-conscious shortenings. Future research should focus on industrial-scale production, sensory evaluation, and market feasibility to support its adoption in commercial food processing.

## Figures and Tables

**Table 1:** Physicochemical properties of TSO, PB, IO, and commercially available shortening A, B, and C.

	SMP (℃)	AV (mg/g)	PV (mmol/kg)	Water Content (%)	Plasticity Ranges (℃)	Whipping Degree (mL/g)	Polyphenol Content (mg/kg)	OSI (h)
PO	32.30 ± 0.04 ^e^	0.44 ± 0.06 ^bc^	2.26 ± 0.07 ^cd^	0.06 ± 0.01 ^bc^	14.00 ± 0.60 ^d^	1.64 ± 0.03 ^cd^	16.01 ± 1.14 ^d^	6.30 ± 0.11 ^c^
TSO	51.40 ± 0.76 ^a^	0.55 ± 0.09 ^b^	2.38 ± 0.09 ^c^	0.05 ± 0.00 ^cd^	15.0 ± 0.50 ^c^	1.53 ± 0.07 ^d^	9.85 ± 0.39 ^f^	6.53 ± 0.06 ^bc^
SO	/	0.43 ± 0.04 ^bc^	2.19 ± 0.04 ^d^	0.04 ± 0.00 ^d^	/	/	20.26 ± 0.20 ^a^	6.48 ± 0.03 ^c^
PB	/	0.56 ± 0.06 ^b^	2.62 ± 0.07 ^b^	0.10 ± 0.01 ^a^	10.50 ± 0.50 ^e^	1.72 ± 0.04 ^bc^	17.23 ± 0.07 ^bc^	5.79 ± 0.11 ^d^
IO	41.30 ± 0.40 ^c^	0.79 ± 0.12 ^a^	2.80 ± 0.03 ^a^	0.06 ± 0.00 ^c^	17.50 ± 0.47 ^b^	1.85 ± 0.11 ^a^	17.32 ± 0.38 ^bc^	6.73 ± 0.12 ^bc^
A	40.60 ± 0.40 ^d^	0.48 ± 0.05 ^bc^	2.56 ± 0.06 ^b^	0.06 ± 0.00 ^c^	15.60 ± 0.60 ^c^	1.90 ± 0.04 ^a^	17.89 ± 0.26 ^b^	6.69 ± 0.10 ^b^
B	42.60 ± 0.15 ^c^	0.35 ± 0.04 ^c^	2.35 ± 0.09 ^c^	0.06 ± 0.01 ^bc^	18.90 ± 0.75 ^a^	1.82 ± 0.03 ^ab^	16.96 ± 0.13 ^c^	7.05 ± 0.16 ^a^
C	39.80 ± 0.42 ^de^	0.49 ± 0.04 ^b^	2.66 ± 0.11 ^ab^	0.08 ± 0.01 ^b^	17.00 ± 0.50 ^b^	1.80 ± 0.08 ^ab^	14.39 ± 0.24 ^e^	6.34 ± 0.21 ^c^

“/” means not detected. Fatty acids containing less than 0.1% are not listed. Values in the table are the mean, standard deviation, and superscript letters indicate significant differences in the same row of data (*p* < 0.05).

**Table 2:** Fatty acid composition of TSO, PB, IO, and commercially available shortening A, B, and C.

	TSO	PO	SO	PB	IO	A	B	C
C12:0	0.88 ± 0.01 ^d^	0.21 ± 0.01 ^f^	/	0.41 ± 0.01 ^e^	0.40 ± 0.03 ^e^	1.57 ± 0.08 ^c^	3.43 ± 0.23 ^a^	2.23 ± 0.12 ^b^
C14:0	0.17 ± 0.01 ^f^	2.25 ± 0.02 ^d^	0.20 ± 0.00 ^f^	0.45 ± 0.05 ^e^	0.45 ± 0.09 ^e^	9.24 ± 0.09 ^b^	11.89 ± 0.31 ^a^	4.24 ± 0.19 ^c^
C16:0	62.84 ± 0.80 ^a^	57.96 ± 0.64 ^b^	11.81 ± 0.09 ^f^	44.42 ± 0.11 ^c^	44.39 ± 0.18 ^c^	36.25 ± 0.16 ^d^	35.59 ± 0.27 ^e^	34.05 ± 0.06 ^e^
C16:1	0.21 ± 0.01 ^d^	0.73 ± 0.01 ^b^	0.18 ± 0.01 ^d^	0.27 ± 0.02 ^c^	0.27 ± 0.01 ^c^	/	/	0.82 ± 0.04 ^a^
C18:0	1.49 ± 0.01 ^e^	6.08 ± 0.09 ^b^	5.43 ± 0.26 ^c^	4.23 ± 0.18 ^d^	4.19 ± 0.15 ^d^	5.25 ± 0.10 ^c^	10.69 ± 0.49 ^a^	6.30 ± 0.16 ^b^
C18:1	31.47 ± 0.21 ^a^	25.99 ± 0.44 ^c^	22.58 ± 0.41 ^d^	26.44 ± 0.32 ^c^	26.48 ± 0.27 ^c^	31.45 ± 0.25 ^a^	29.25 ± 0.09 ^b^	29.17 ± 0.07 ^b^
C18:2	2.27 ± 0.09 ^f^	5.40 ± 0.16 ^e^	53.85 ± 0.08 ^a^	21.14 ± 0.18 ^b^	21.14 ± 0.07 ^b^	11.14 ± 0.08 ^c^	7.26 ± 0.17 ^d^	21.13 ± 0.07 ^b^
C18:3	0.17 ± 0.00 ^e^	0.25 ± 0.02 ^de^	5.19 ± 0.06 ^a^	2.22 ± 0.10 ^c^	2.20 ± 0.17 ^c^	2.65 ± 0.12 ^b^	0.38 ± 0.01 ^d^	0.38 ± 0.01 ^d^
C20:0	0.16 ± 0.00 ^e^	0.32 ± 0.01 ^b^	0.25 ± 0.00 ^cd^	0.12 ± 0.01 ^f^	0.12 ± 0.00 ^f^	1.24 ± 0.12 ^a^	0.23 ± 0.01 ^de^	0.32 ± 0.01 ^bc^
ΣSFA	65.53 ± 0.29 ^b^	66.84 ± 0.62 ^a^	17.68 ± 0.34 ^f^	49.63 ± 0.26 ^e^	49.55 ± 0.19 ^e^	53.54 ± 0.06 ^d^	61.83 ± 0.24 ^c^	47.13 ± 0.13 ^e^
ΣUSFA	34.12 ± 0.20 ^f^	32.36 ± 0.33 ^f^	80.80 ± 0.33 ^a^	50.07 ± 0.13 ^c^	50.08 ± 0.24 ^c^	45.24 ± 0.08 ^d^	36.89 ± 0.23 ^e^	51.51 ± 0.07 ^b^
ΣTFA	/	/	/	/	/	0.65 ± 0.14 ^a^	0.27 ± 0.02 ^b^	0.15 ± 0.03 ^b^

“/” means not detected. Fatty acids containing less than 0.1% are not listed. Values in the table are the mean ± standard deviation, and superscript letters indicate significant differences in the same row of data (*p* < 0.05).

## Data Availability

The original contributions presented in the study are included in the article, further inquiries can be directed to the corresponding author.
